# Microbial diversity and their extracellular enzyme activities among different soil particle sizes in mossy biocrust under N limitation in the southeastern Tengger Desert, China

**DOI:** 10.3389/fmicb.2024.1328641

**Published:** 2024-01-29

**Authors:** Xiaomin Duan, Jiajia Li, Wangping He, Jingjing Huang, Wanxiang Xiong, Shijia Chi, Siyuan Luo, Jianli Liu, Xiu Zhang, Jingyu Li

**Affiliations:** ^1^College of Biological Science and Engineering, North Minzu University, Yinchuan, China; ^2^Ningxia Key Laboratory of Microbial Resources Development and Applications in Special Environment, Yinchuan, China; ^3^Key Laboratory of Ecological Protection of Agro-pastoral Ecotones in the Yellow River Basin, National Ethnic Affairs Commission of the People’s Republic of China, Yinchuan, China

**Keywords:** mossy biocrust, rhizoid soil, bacterial diversity, fungal diversity, extracellular enzyme activities, N limitation

## Abstract

**Introduction:**

Mossy biocrust represents a stable stage in the succession of biological soil crust in arid and semi-arid areas, providing a microhabitat that maintains microbial diversity. However, the impact of mossy biocrust rhizoid soil and different particle sizes within the mossy biocrust layer and sublayer on microbial diversity and soil enzyme activities remains unclear.

**Methods:**

This study utilized Illumina MiSeq sequencing and high-throughput fluorometric technique to assess the differences in microbial diversity and soil extracellular enzymes between mossy biocrust rhizoid soil and different particle sizes within the mossy biocrust sifting and sublayer soil.

**Results:**

The results revealed that the total organic carbon (TOC), total nitrogen (TN), ammonium (NH_4_^+^) and nitrate (NO_3_^−^) in mossy biocrust rhizoid soil were the highest, with significantly higher TOC, TN, and total phosphorus (TP) in mossy biocrust sifting soil than those in mossy biocrust sublayer soil. Extracellular enzyme activities (EAAs) exhibited different responses to various soil particle sizes in mossy biocrust. Biocrust rhizoid soil (BRS) showed higher C-degrading enzyme activity and lower P-degrading enzyme activity, leading to a significant increase in enzyme C: P and N: P ratios. Mossy biocrust soils were all limited by microbial relative nitrogen while pronounced relative nitrogen limitation and microbial maximum relative carbon limitation in BRS. The diversity and richness of the bacterial community in the 0.2 mm mossy biocrust soil (BSS_0.2_) were notably lower than those in mossy biocrust sublayer, whereas the diversity and richness of the fungal community in the rhizoid soil were significantly higher than those in mossy biocrust sublayer. The predominant bacterial phyla in mossy biocrust were Actinobacteriota, Protebacteria, Chloroflexi, and Acidobacteriota, whereas in BSS_0.2_, the predominant bacterial phyla were Actinobacteriota, Protebacteria, and Cyanobacteria. Ascomycota and Basidiomycota were dominant phyla in mossy biocrust. The bacterial and fungal community species composition exhibited significant differences. The mean proportions of Actinobacteriota, Protebacteria, Chloroflexi, Acidobacteriota, Acidobacteria, Cyanobacteria, and Bacteroidota varied significantly between mossy biocrust rhizoid and different particle sizes of mossy biocrust sifting and sublayer soil (*p* < 0.05). Similarly, significant differences (*p* < 0.05) were observed in the mean proportions of Ascomycota, Basidiomycota, and Glomeromycota between mossy biocrust rhizoid and different particle sizes within the mossy biocrust sifting and sublayer soil. The complexity and connectivity of bacterial and fungal networks were higher in mossy biocrust rhizoid soil compared with different particle sizes within the mossy biocrust sifting and sublayer soil.

**Discussion:**

These results offer valuable insights to enhance our understanding of the involvement of mossy biocrust in the biogeochemical cycle of desert ecosystems.

## 1 Introduction

Mossy biocrusts represent the most stable successional stage of biological soil crust (Lan et al., 2015). Their rhizoids penetrate deep into the sand layer and encapsulate soil particles through secretions, rhizoids, and mycelium, effectively stabilizing the sand dunes (Lan et al., 2011; Yang et al., 2022). These biocrusts play a crucial role in regulating soil hydrology, increasing soil organic matter, sequestering atmospheric carbon and nitrogen, sustaining biodiversity in desert ecosystems, and supporting plant fixation (Pietrasiak et al., 2013; Belnap et al., 2014; Yang et al., 2020; Li et al., 2023). Recent studies have also revealed that the rhizoid of mossy biocrust possess the functions of higher plants, including the absorption and transfer of soil nutrients, making them an integral biological component of desert ecosystems (Yang et al., 2022).

The organic matter content in the mossy biocrust layer surpasses that found in the biocrust sublayer, bare sand, and biocrust at various stages of development (Gao et al., 2017; Xu et al., 2022). Mossy biocrusts have demonstrated the capacity to enhance soil development and refine soil particles (Esteban et al., 2014; Yang et al., 2022). It has been observed that the soil particles in different types of biocrust are predominantly composed of fine sand (Esteban et al., 2014). Furthermore, alterations in the soil particle composition are contingent upon the interception of fine particles by the crust (Six et al., 2004). Soil particles with different particle sizes constitute the micro and macro structure of soil aggregates (Hemkemeyer et al., 2015; Rabbi et al., 2015), directly influencing soil moisture, fertility, air, and heat, regulating soil nutrients, and impacting the soil microbial community (Srivastava et al., 2020). The soil aggregates of different particle sizes are influenced by soil environmental factors, such as pH, organic carbon, carbon–nitrogen ratio, water content, and total nitrogen, subsequently affecting the soil microbial community (Sessitsch et al., 2001; Hemkemeyer et al., 2018). Given the limited existing literature on the rhizoid of mossy biocrust, both domestically and internationally, it is imperative to comprehend the structure and function of the microbial community within the mossy biocrust rhizoid soil. This will contribute to understanding the role and function of soil microbes in the sustainable development of ecosystems (Rabbi et al., 2015; Ling et al., 2022).

Soil microorganisms produce a variety of extracellular enzymes that drive the circulation and transformation of carbon, nitrogen, and phosphorus in the soil (Yang et al., 2020; Sokol et al., 2022). Soil enzymes play a vital role in soil metabolism (Sinsabaugh et al., 2008). However, only a few soil enzymes have the capability to catalyze terminal hydrolysis reactions (Cui et al., 2023). For instance, soil urease is crucial in the soil nitrogen cycle, soil β-glucosidase facilitates carbon acquisition, and soil phosphatase contributes to phosphorus mineralization (Xu et al., 2022). Extracellular enzyme activities (EEAs) directly influence the effectiveness of soil nutrient, and the stoichiometry of extracellular enzyme activities is considered a valid indicator of microbial metabolism for resource utilization (Zhu et al., 2020). Microbial communities play a role in regulating the carbon and nitrogen cycles of biological soil crust in desert ecosystems. Different microorganisms and soil particles create diverse microbial environments, wherein bacteria secrete biopolymers as adhesives for soil particles, and fungi can bind to soil particles through mycelium. However, most of the existing studies have not distinguished between the rhizoid soil of mossy biocrust and different particle sizes in mossy biocrust layer and sublayer. Recent research on biocrust in arid and semi-arid regions has primarily focused on the biocrust layer and sublayer during different succession periods, while less research has been conducted on mossy biocrust rhizoid soil and different particle sizes in mossy layer and sublayer soil, with limited exploration of mossy biocrust rhizoid soil and different particle sizes within mossy layer and sublayer. Therefore, it is important to investigate how extracellular enzyme activity, soil content, and microbial community structure differ between the rhizoid soil of mossy biocrust and the soil of different particle sizes within the mossy biocrust layer and sublayer.

Therefore, we hypothesized that the organic matter content of mossy biocrust rhizoid soil is higher than that of mossy biocrust sifting soil and mossy biocrust sublayer soil. Additionally, we anticipate that the extracellular enzyme activities of mossy biocrust rhizoid soil will be higher than those of different soil particle sizes in mossy biocrust sifting soil and mossy biocrust sublayer soil. Finally, we expect the microbial community structure of the rhizoid soil of mossy biocrust to differ from that of different soil particle sizes in mossy biocrust sifting soil and mossy biocrust sublayer soil. To test this hypothesis, we analyzed the microbial diversity of mossy biocrust rhizoid soil and different particle sizes in mossy biocrust sifting soil and mossy biocrust sublayer soil in the southeastern Tengger Desert using Illumina MiSeq technology. We analyzed the utilization of soil nutrients by microorganisms in different particle-size soils of the mossy biocrust by measuring extracellular enzyme activity. The main research questions of our project were as follows: (1) Whether the organic matter content and enzyme activity of mossy biocrust rhizoid soil are significantly different from those different soil particle sizes in mossy biocrust sifting soil and mossy biocrust sublayer soil. (2) What are the similarities and differences in the structural composition of the microbial community in mossy biocrust rhizoid soil compared with mossy biocrust sifting soil and mossy biocrust sublayer soil? We aimed to investigate the impact of mossy biocrust rhizoid soil, as well as the mossy biocrust layer and sublayer soil, on microbial community structure, diversity, and soil extracellular enzyme activity.

## 2 Materials and methods

### 2.1 Study sites

The sampling sites were situated at the southeastern edge of the Tengger Desert, Ningxia Hui Autonomous Region (37°2′,104°5′). This region experiences an annual average temperature of 10.0°C, with low-temperature extremes reaching −25.1°C and high-temperature extremes reaching 38.1°C; the area receives an annual average of 3,264 sunshine hours and a mean precipitation of 186 mm per year (Li et al., 2012). The region is characterized by an annual potential evaporation of 3,000 mm, an average annual wind speed of 2.9 m/s, and an average of 59 days of sandstorm (Li et al., 2020). In 1956, the Chinese Academy of Sciences and related institutions established a 16 km long and 0.5 km wide vegetation protection corridor in the Shapotou area, to facilitate the smooth passage of the Baolan Railway through the dunes. After 60 years of implementation, the stabilization zone has expanded, the mobile dunes have stabilized, and the deposition of dust has resulted in the development of biological soil crusts. Additionally, a significant number of cryptogamic plants, such as algae, mosses, and lichens, have colonized the area and contributed to the fixation of the sand surface (Lan et al., 2011).

### 2.2 Soil sampling and treatment

On 20 June 2022, we conducted random selection of five 5*5 meter mossy biocrust quadrats in the Shapotou area. Utilizing the five-point sampling method, we placed the mossy biocrust and sublayer into sterile bags and carefully labeled them. From each quadrat, we collected five mossy biocrust soil samples (each sub-sample approximately 10 cm*10 cm in size) and five mossy biocrust sublayer soil samples, resulting in 50 samples. The samples were placed in an ice box and transported back to the laboratory.

Subsequently, the mossy biocrust sublayer soil from each sample was passed through 1 mm, 0.5 mm, and 0.2 mm sieves, and the resulting materials were mixed together. The sub-samples that passed the 0.2 mm sieve were combined and labeled as biocrust sublayer (BS) soils, those that on the 0.2 mm sieve were designated as BS_0.2_, and those that on the 0.5 mm sieve were identified as BS_0.5_.

Due to the small and densely intertwined nature of the rhizomes in mossy biocrust, it was challenging to collect rhizoid soil. The process involved placing the mossy biocrust layer in a 20*30 cm enamel tray and breaking it up with a fine dissecting needle. The moss was then carefully separated using sharp-nosed forceps and sifted using 1 mm, 0.5 mm, and 0.2 mm sieves. The small and fragile nature of the moss rhizoid made it susceptible to breakage, hence the moss plants on the 1 mm sieve were collected, transferred to a 50 mL centrifuge tube containing PBS (phosphate buffer solution), and subjected to shaking at 120 rpm/min for 10 min at room temperature (Beckers et al., 2016). Subsequently, the soil suspension in the centrifuge tube was filtered through a sterile filter into a clean 50mL centrifuge tube and then centrifuged at 4 °C and 6,000g for 20 minutes. The resulting supernatant was discarded, leading to the isolation of the rhizoid soil samples, designated as BRS (biocrust rhizoid soil), with a portion promptly frozen using liquid nitrogen in an -80 °C ultra-low temperature refrigerator. The soil that passed through a 0.2 mm sieve is referred to as mossy biocrust sublayer soil (BSS). Additionally, the biocrust sifting soil on 0.2 mm and 0.5 mm sieves was amalgamated and designated as BSS_0.2_. All samples were stored at −20°C for further experiments, while the remaining samples air-dried in the laboratory for the determination of physical and chemical properties of soil. Due to the limited number of larg-sized soil samples obtained, only the physical and chemical properties of BRS, BSS, and BS soils were determined, and a portion of all samples was stored at 4°C for experiments, focusing on extracellular enzyme activities.

### 2.3 Soil chemistry and extracellular enzyme analyses

Soil nitrate nitrogen (NO_3_^−^) was determined by ultraviolet spectrophotometer (Moorcroft et al., 2001), soil ammonium nitrogen (NH_4_^+^) was determined via indophenol blue colorimetric method (Dorich and Nelson, 1983), soil total nitrogen (TN) was analyzed using the Kjeldahl method (Zhang et al., 2015), soil total phosphorus (TP) was determined by NaOH melting-molybdenum antimony resistance colorimetry (Zhang et al., 2015), and soil organic C (TOC) was measured using potassium dichromate redox titration (Eyherabide et al., 2014).

The activity of C-degrading enzymes (β-1,4-glucosidase, and BG), two N-degrading enzymes (leucine aminopeption, LAP and β-1,4-N-acetylglucosaminidase, and NAG), and P-degrading enzymes (acid phosphatase, and AP) in BS, BS_0.2_, BS_0.5_, BSS, BSS_0.2_, and BRS was measured. These EEAs were assessed using standard fluorometric techniques with highly fluorescent compounds, such as 7-amino-4-methylcoumarin and 4-methylumbelliferone (Sinsabaugh et al., 2008). Soil enzyme activities were conducted within 1 week of sample treatment. In total, 2.00 g of treated soil was suspended in 125 mL of 50 mM sodium acetate buffer (pH = 5.0) in a vortex oscillator for 1 min to obtain soil suspension. Black 96-well microplates were used for fluorescence analysis, with sample determination, sample control, quenching control, reference standard, negative controls, and blank wells, each with eight duplicate wells. The enzyme activity assays were performed according to the experimental procedure outlined by Zhu et al. (2020); the extracellular enzymes are expressed as nmol MU g^−1^ soil (dry weight) h^−1^. The stoichiometry of the extracellular enzymes C: N ratio, C: P ratio, and N: P ratio was expressed as Ln (BG): Ln (LAP + NAG), Ln (BG): Ln (AP), and Ln (LAP + NAG): Ln (AP). The soil enzyme activity (Ab) is calculated using the following equations:


$$\mathrm{Ab}=FV/\left(\mathrm{eV}1tm\right)$$

In this formula, F is the corrected fluorescence value of the soil sample; V is the total volume of soil suspension (125 mL), V1 is the soil suspension volume of each hole in the 96-well microtiter plate (0.2 mL), t is the incubation time (4 h), and m is the dry weight of 2 g of wet soil.


$$F=\left(f-fb\right)/q-\mathrm{fs}$$

In this formula, f denotes the average of the count of the reader of the fluorescence assay wells, fb is the average of the count of the reader of the fluorescence sample control wells, q is the hardening coefficient, and fs is the average value of the fluorescence negative control wells.


$$e=fr/\left(cV2\right)$$

In this formula, e is the fluorescence release coefficient, fr is the average value of fluorescence reference standard wells, c is the content of the reference standard well (10 μM), and V2 is the volume of the reference standard (50 μL).


$$q=\left(fq-fb\right)/fr$$

In this formula, q is the hardening coefficient, fq is the average fluorescence value of the quench control, fb is the average of the count of the reader of the fluorescence sample control wells, and fr is the average value of fluorescence reference standard wells.

The enzyme measurement vector model was used to calculate the microbial metabolic limitation (Moorhead et al., 2013, 2016). The formula is as follows:


$$\mathrm{microbial}\;\mathrm{relative}\;C\;\mathrm{limitation}:\mathrm{Length}=\surd \left(x2+y2\right)$$

$$\mathrm{microbial}\;N\;\mathrm{or}\;P\;\mathrm{limitation}:\mathrm{Angle}={}^{\circ}\mathrm{Atan}2\left(x,y\right)$$

$$\mathrm{where}\;x=BG/\left(BG+AP\right),y=BG/\left(BG+\mathrm{LAP}+\mathrm{NAG}\right)$$

In the formula, the longer the vector length, the greater the relative C limitation of microorganisms; the vector angle greater than 45° indicates that microorganisms are affected by soil P limitation, the angles less than 45° indicate that microorganisms are restricted by soil N limitation. The microbial P limitation increases with increasing the angle, while the N limitation increases with decreasing the angle.

### 2.4 DNA extraction, PCR amplification, and Illumina sequencing

Soil DNA was extracted according to instructions of DNeasy®96 PowerSoil®Pro QIAcube®HT Kit, and extracted genomic DNA was detected by 1% agarose gel electrophoresis.

338F/806R were used to amplify the V3-V4 region of bacterial 16S rDNA (Liu et al., 2019), and ITS1F/ITS2R were used to amplify the fungal ITS region (Zhang et al., 2022). The PCR amplification reaction system of bacterial 16S rDNA V3-V4 region had 20 μL of mixture including 4.0 μL 5*Fastpfu Buffer, 2.0 μL 2.5 mM dNTPs, 0.8 μL of each primer (5 μM), 10 ng template DNA, 0.4 μL FastPfu Polymerase, and 0.2 μL BSA, finally added to 20 μL ddH_2_O. The PCR amplification reaction system of fungal ITS region had 20 μL of mixture including 2.0 μL 10* Buffer, 2.0 μL 2.5 mM dNTPs, 0.8 μL of each primer (5 μM), 10 ng template DNA, 0.2 μL rTap polymerase, and 0.2 μL BSA, finally added to 20 μL ddH_2_O. The cycling program was 95°C for 3 min, 95°C for 30 s, 55°C for 30 s, 72°C for 45 s, (bacteria 27 cycles; fungi 35 cycles), and a final extension at 72°C for 10 min. Each sample was tested with three replicates, three replicates of PCR products were mixed and detected by electrophoresis using a 2% agarose gel.

The PCR products were recovered by gel cutting using AXYGEN’s AxyPrepDNA Gel Recovery Kit, and the PCR products were quantified using the QuantiFluor™ -ST Blue Fluorescence Quantification System (Promega company) and then mixed in equal proportions according to the amount of sequencing required for each sample; the PCR products were used to build a library using the NEXTFLEX® Rapid DNA-Seq Kit. Sequencing was performed using the HiSeq 2000 high-throughput sequencing device, according to the standard experimental procedure provided by Illumina. Sequences were deposited into the NCBI Sequence Read Archive under accession number SRP438853.

### 2.5 Statistical analyses

Physicochemical properties and the differences between soil samples were analyzed using one-way analysis of variance (Tukey’s HSD *p* < 0.05) in GraphPad Prism5 software. Additionally, one-way ANOVA was utilized to determine the significance of the difference in enzyme activity between soil samples, followed by Tukey’s test for multiple comparisons (*p* < 0.05). The raw sequencing data underwent quality control using Trimmomatic software, followed by merging the ends using FLASH software. The optimized sequences after quality control and splicing were noise-reduced using the DADA2 plug-in within the QIIME2 process (default parameters), with the exception of PCR amplification errors or sequencing errors present in the optimized data. The amplified sequence variants (ASVs) were subjected to separate taxonomic analysis of bacterial and fungal species using the Naive Bayes classifier in QIIME2, with a confidence score of 0.7 and clustering sequences with 99% similarity to one ASV. To facilitate downstream diversity and composition analysis, the sequence level of each sample was standardized to the minimum number of sample sequences. Specifically, bacterial samples had 11,912 sequences per sample, resulting in 7,453 ASVs, while fungi were standardized to 38,153 valid sequences per sample, resulting in 2,947 ASVs. The dilution curves of high-throughput sequencing for bacterial and fungal samples exhibited sequencing depth. All analysis processes were conducted through the cloud platform diversity analysis process (QIIME2 process) provided by Shanghai Majorbio Bio-Pharm Technology Co. Ltd., culminating in the annotation of ASV information at phylum and genus levels.

Bacterial and fungal community richness and diversity across different treated soil samples were examined by calculating the Chao1 index and the Shannon index (reflecting the alpha diversity of microbial communities). Principal component analysis (PCoA) using the Bray–Curtis distance matrix was performed via the R language Vegan package, to assess the differences between samples. The interpretation of sample differences by different grouped factors was analyzed, with statistical significance assessed through the permutation test. Additionally, the Stat package of R language was utilized to test the difference between groups by examining the top 15 genera, evaluate the significance level of species abundance difference, and obtain the species information with significant differences between two groups and multiple groups. Furthermore, network co-occurrence maps were constructed to investigate reciprocal associations between microbial ASV levels, wherein ASV with less than 0.1% abundance were combined before constructing the network co-occurrence map; the threshold was determined by calculating the Spearman correlation coefficient and the Jaccard distance, with *p*-values being corrected using the false discovery rate (Rottjers and Faust, 2018; Yuan et al., 2021; Guang et al., 2023). Interactivity of the graphical representation was achieved using the Hminimal” and “igraph” packages of R language “and the Gephi visualization platform; positive correlations were represented by red edges, while negative correlations were denoted by green edges in the co-occurrence network graph.

## 3 Results

### 3.1 Chemical characteristics of sample soils

**Figure 1:**
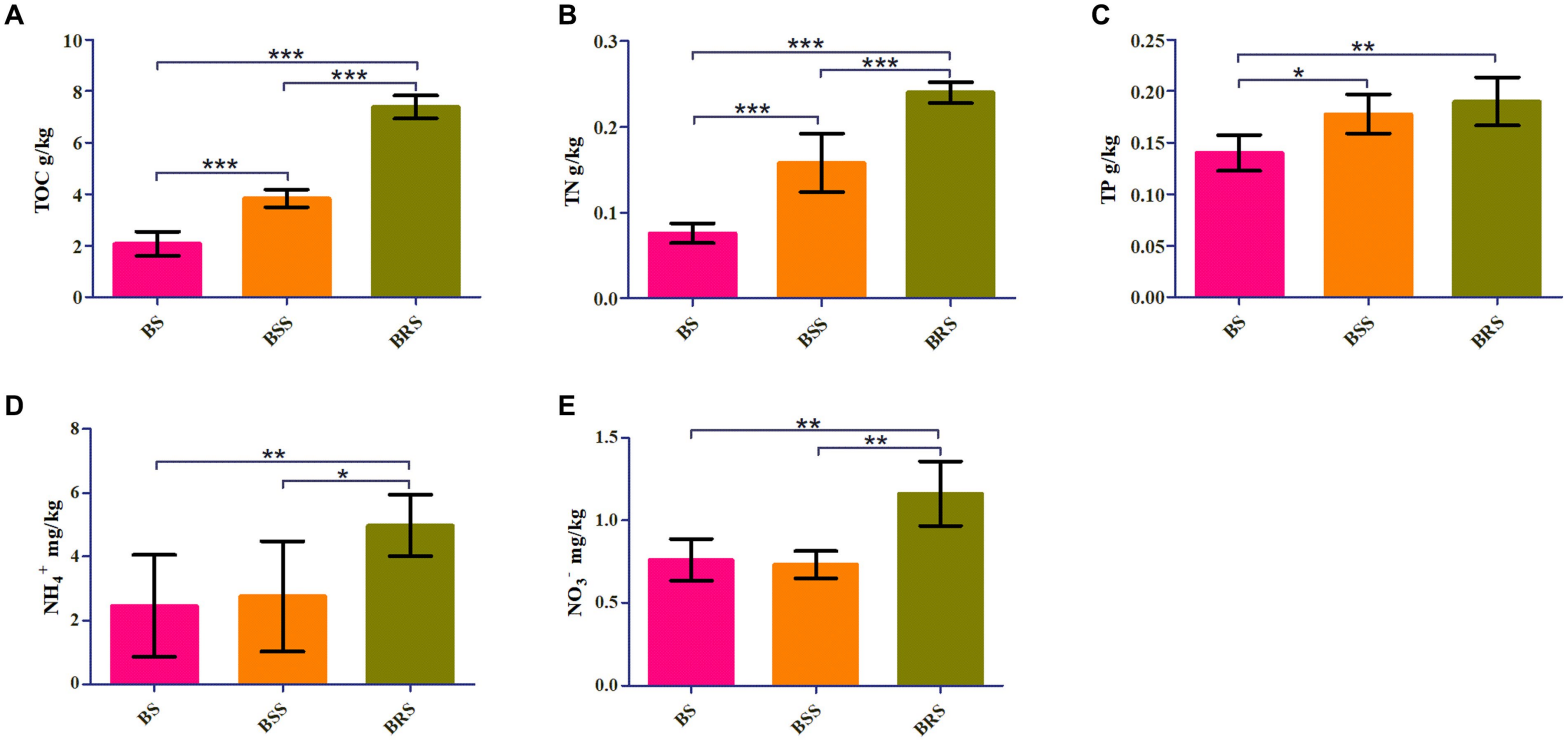
Chemical characteristics of soil. Comparative analysis of chemical factors in mossy biocrust rhizoid soil, mossy biocrust sifting soil, and mossy biocrust sublayer soil **(A–E)**. BSS indicated mossy biocrust layer soil (BSS_0.2_ and BSS), BS mossy biocrust sublayer soil (BS_0.5_, BS_0.2_, and BS), and BRS indicated mossy biocrust rhizoid soil. TOC, total organic carbon. TN, total nitrogen. TP, total phosphorus. NH_4_^+^, ammonium nitrogen. NO_3_^−^, nitrate nitrogen. BSS, mossy biocrust sifting soil with particle size less than 0.2 mm. BSS_0.2_, mossy biocrust sifting soil with particle size greater than 0.2 mm and less than 1 mm. BRS, mossy biocrust rhizoid soil with particle size of less than 0.3 mm. BS, mossy biocrust sublayer soil with particle size less than 0.2 mm. BS_0.2_, mossy biocrust sublayer soil with particle size greater than 0.2 mm and less than 0.5 mm. BS_0.5_, mossy biocrust sublayer soil with particle size greater than 0.5 mm and less than 1 mm. BS, mossy biocrust sublayer soil with particle size of less than 0.2 mm.

TOC, TN, and NO_3_^−^ were significantly higher in BRS compared with BS and BSS (*p* < 0.01). Additionally, NH_4_^+^ and NO_3_^−^ were significantly higher in BRS than that in BS and BSS (*p* < 0.05). Furthermore, TP was significantly higher in BRS than in BS (*p* < 0.01). Moreover, The TOC and TN of BSS were significantly higher than those of BS, while TP in BSS was significantly higher than in BS (*p* < 0.05; Figure 1).

### 3.2 Microbial enzyme activities and metabolic restriction

**Figure 2:**
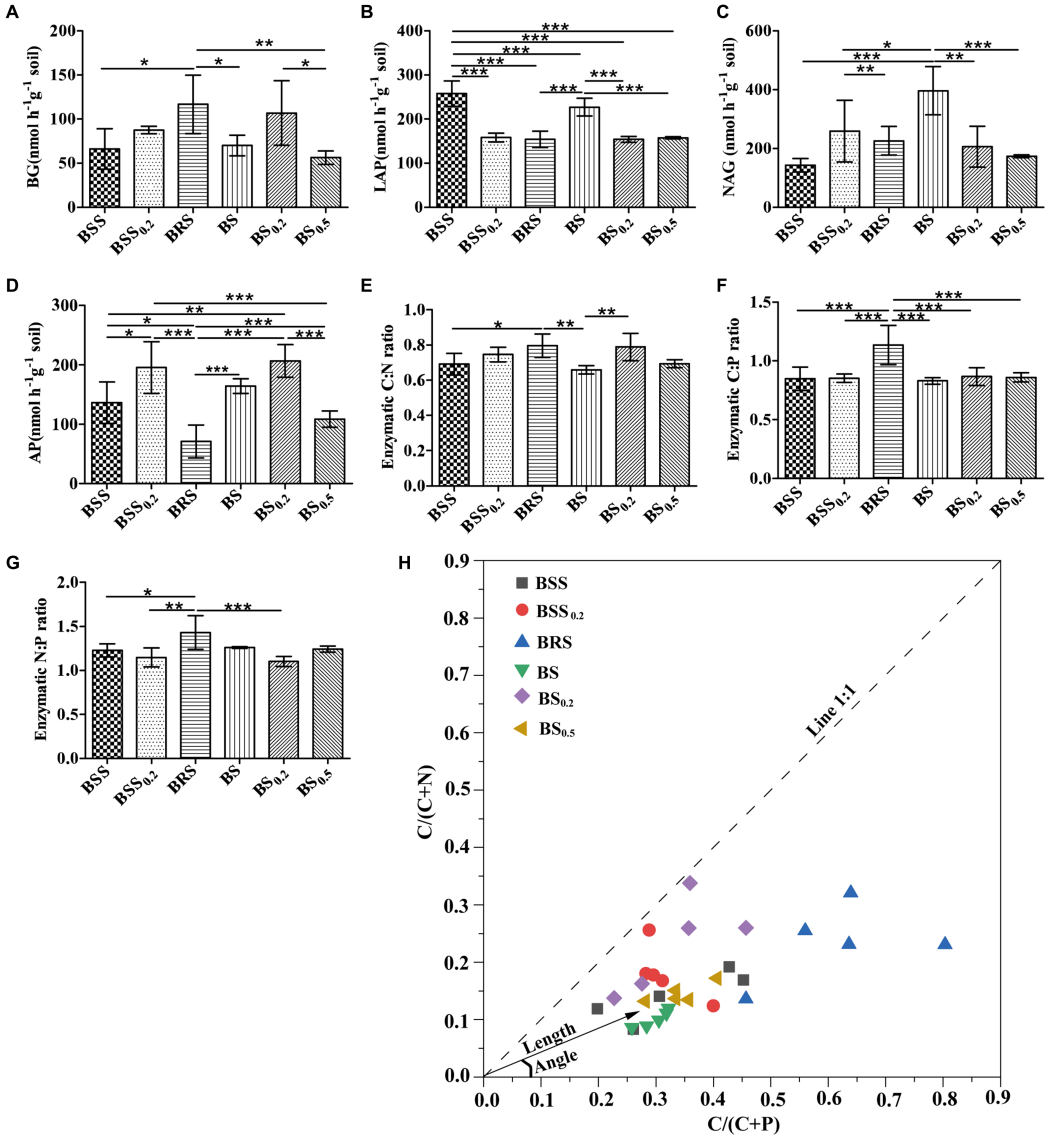
Stoichiometry of soil extracellular enzymes and microbial nutrient limitation. C-, N-, and P-degrading enzymes **(A–D)** and their stoichiometric ratios **(E–G)** and microbial nutrient limitation **(H)** in six treated soil samples. The meanings of BS_0.5_, BS_0.2_, BS, BSS, BSS_0.2_, and BRS are shown in Figure 1. * showed *p* < 0.05, ** *p* < 0.01, and *** *p* < 0.001.

The EEAs and enzyme stoichiometric ratios exhibited significant difference in mossy biocrust. Specifically, the C-degrading enzyme activity (BG) of BRS was notably higher than that of BSS, BS, and BS_0.5_ (Figure 2A). Furthermore, the enzyme activities of LAP and NAG in BS were significantly higher than BS_0.2_ and BS_0.5_ (Figures 2B,C; *P* < 0.05). In addition, the N-degrading enzyme (LAP) of BSS was the highest (Figure 2B; *P* < 0.05), while the enzyme activities of AP in BRS were the lowest (Figure 2D; *P* < 0.05). Moreover, the enzyme C: N ratio of BRS was significantly higher than that of BSS and BS (Figure 2E), and the enzyme C: P ratio of BRS was the highest (Figure 2F). Additionally, the enzyme N: P ratio of BRS was significantly higher than that of BSS, BSS_0.2_, and BS_0.5_ (Figure 2G).

All data points (Figure 2H) were distributed in the lower right of the 1:1 diagonal, indicating that the mossy biocrust soils were all limited by microbial relative N. Furthermore, the vector length of BRS was the longest, and the included angle of BRS was the largest (Table 1; Figure 2H; *P* < 0.05), suggesting that microorganisms in BRS were limited by microbial relative N and maximum microbial relative C.

**Table 1:** The vector length and angle of the enzyme vector model.

	BSS	BSS_0.2_	BRS	BS	BS_0.2_	BS_0.5_
Angle	22.281 ± 1.9°bc	27.423 ± 7.0°ab	19.805 ± 1.9°bc	18.090 ± 0.5°c	30.629 ± 1.8°a	21.899 ± 1.5°bc
Length	0.358 ± 0.05b	0.368 ± 0.02b	0.664 ± 0.01a	0.314 ± 0.00b	0.409 ± 0.05b	0.372 ± 0.02b

Different lowercase letters indicate significant differences between different soil (*P* < 0.05).

### 3.3 Microbial diversity and composition in mossy biocrust

**Figure 3:**
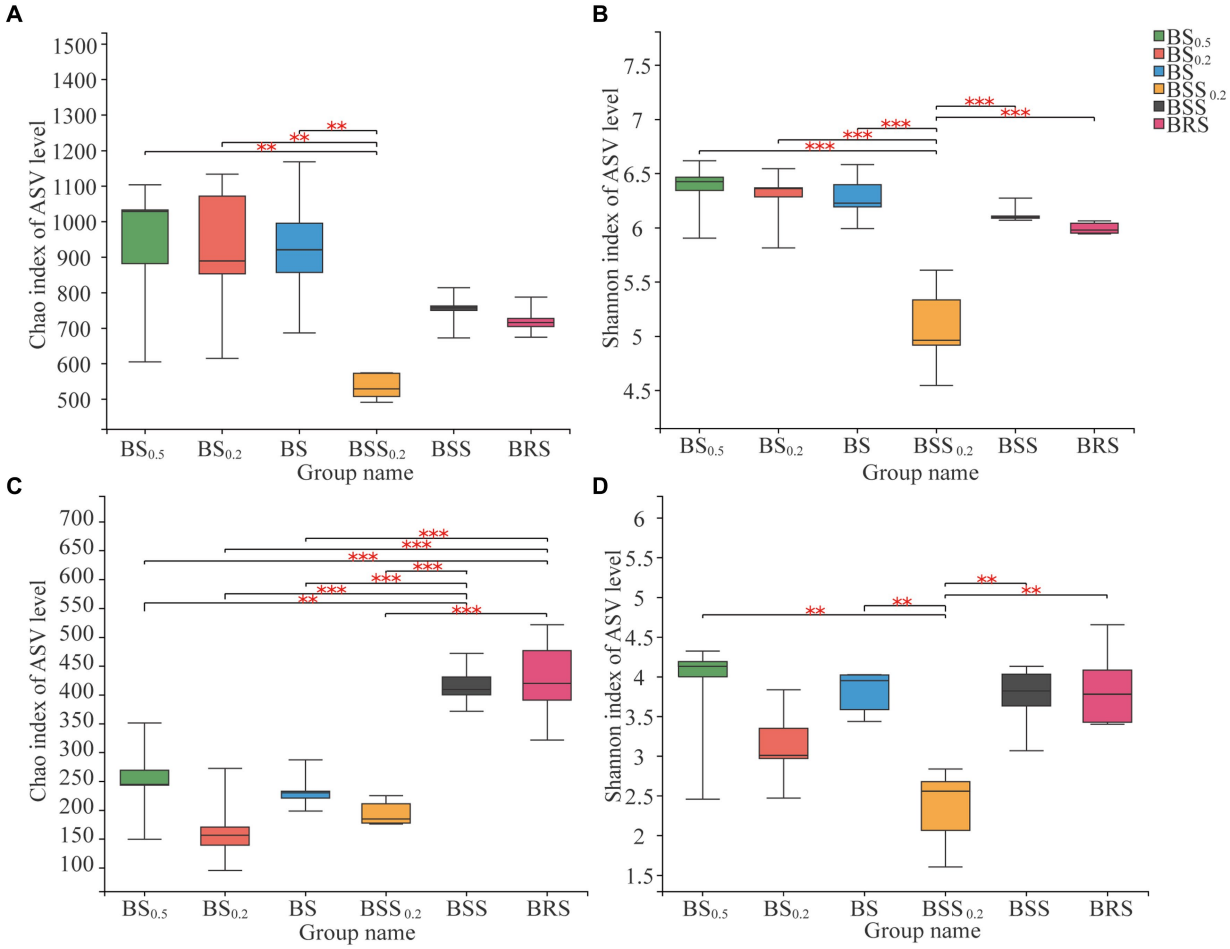
Alpha diversity of microbial communities. **(A)** Chao index of bacterial communities. **(B)** Shannon index of bacterial communities. **(C)** Chao index of fungal communities. **(D)** Shannon index of fungal communities. The meanings of BS_0.5_, BS_0.2_, BS, BSS, BSS_0.2_, and BRS are shown in Figure 1. * showed *p* < 0.05, ** *p* < 0.01, and *** *p* < 0.001.

The bacterial alpha diversity (Chao 1 index) of BBS_0.2_ was significantly lower (*p* < 0.01) than that in BS_0.5_, BS_0.2_, and BS (Figures 3A,B), and the Shannon index of BBS_0.2_ was significantly lower than that in BS_0.5_, BS_0.2_, BS, BBS, and BRS (*p* < 0.01); among them, BS_0.5_, BS_0.2_, and BS belong to the biocrust sublayer, indicating that the richness and diversity of bacterial community in BBS_0.2_ were significantly lower than that in mossy biocrust sublayer with different particle sizes. Additionally, the fungal alpha diversity (Chao 1 index) of BSS and BRS was significantly higher (*p* < 0.01) than that in the biocrust sublayer soil (BS_0.5_, BS_0.2_, and BS) (Figure 3C), indicating that the richness of fungal community in BRS was significantly higher than that in the mossy biocrust sublayer soil; the fungal alpha diversity (Shannon index) of BSS_0.2_ was significantly lower (*p* < 0.01) than that in BS_0.5_, BS, BSS, and BRS (Figure 3D).

**Figure 4:**
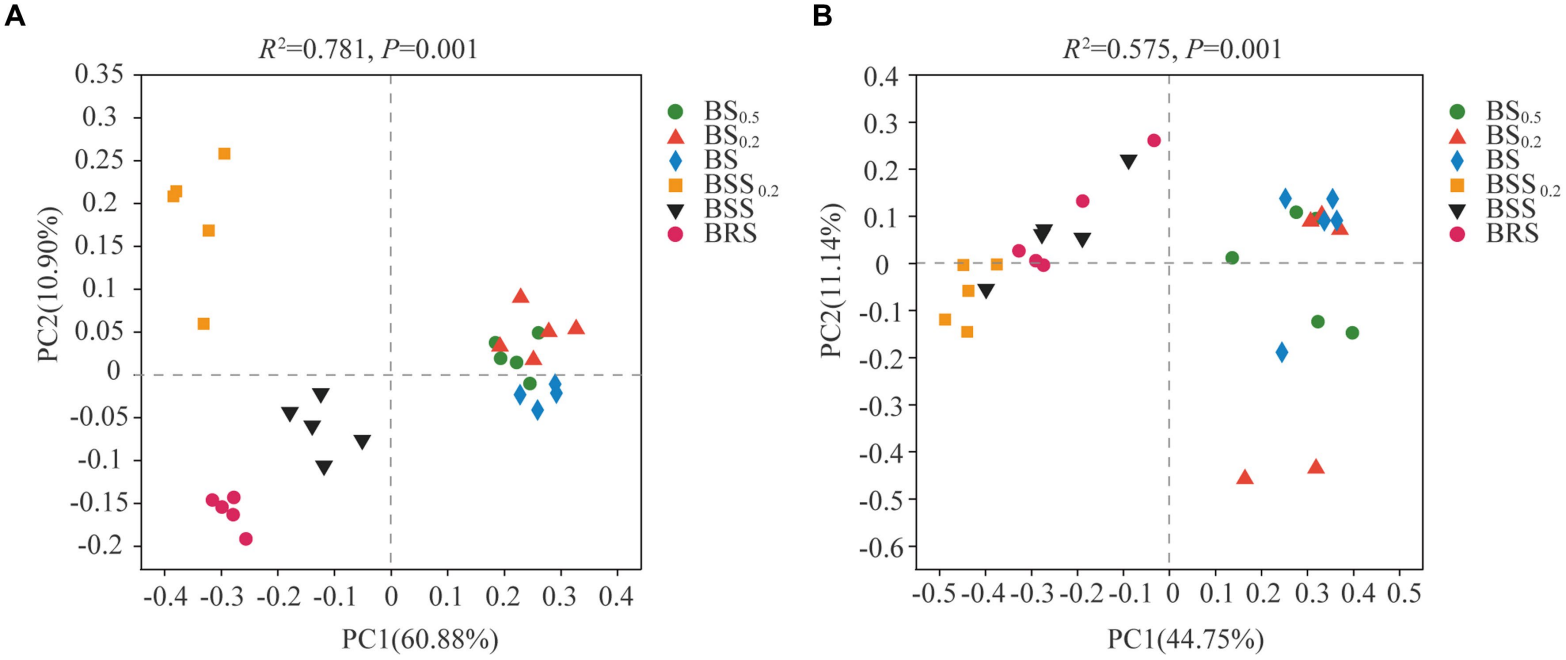
Principal coordinate analysis (PCoA) of bacteria and fungi. **(A)** Bacteria. **(B)** Fungi. The meanings of BS_0.5_, BS_0.2_, BS, BSS, BSS_0.2_, and BRS are shown in Figure 1.

The PCoA results of bacterial and fungal communities at the ASV levels (Figure 4) revealed that the bacterial community structure could be categorized into four groups (Figure 4A): BRS, BSS_0.2_, BSS, and biocrust sublayer soil (BS_0.5_, BS _0.2_, and BS). The bacterial community composition of BRS, BSS_0.2_, BSS, and mossy biocrust sublayer exhibited significant differences. Conversely, the fungal community structure could be roughly divided into two groups: mossy biocrust sublayer (BS_0.5_, BS_0.2_, and BS) and mossy biocrust (BBS _0.2_, BBS, and BRS), and the fungal community composition of mossy biocrust sublayer and mossy biocrust layer also showed significant differences.

### 3.4 Microbial community structure in mossy biocrust

**Figure 5:**
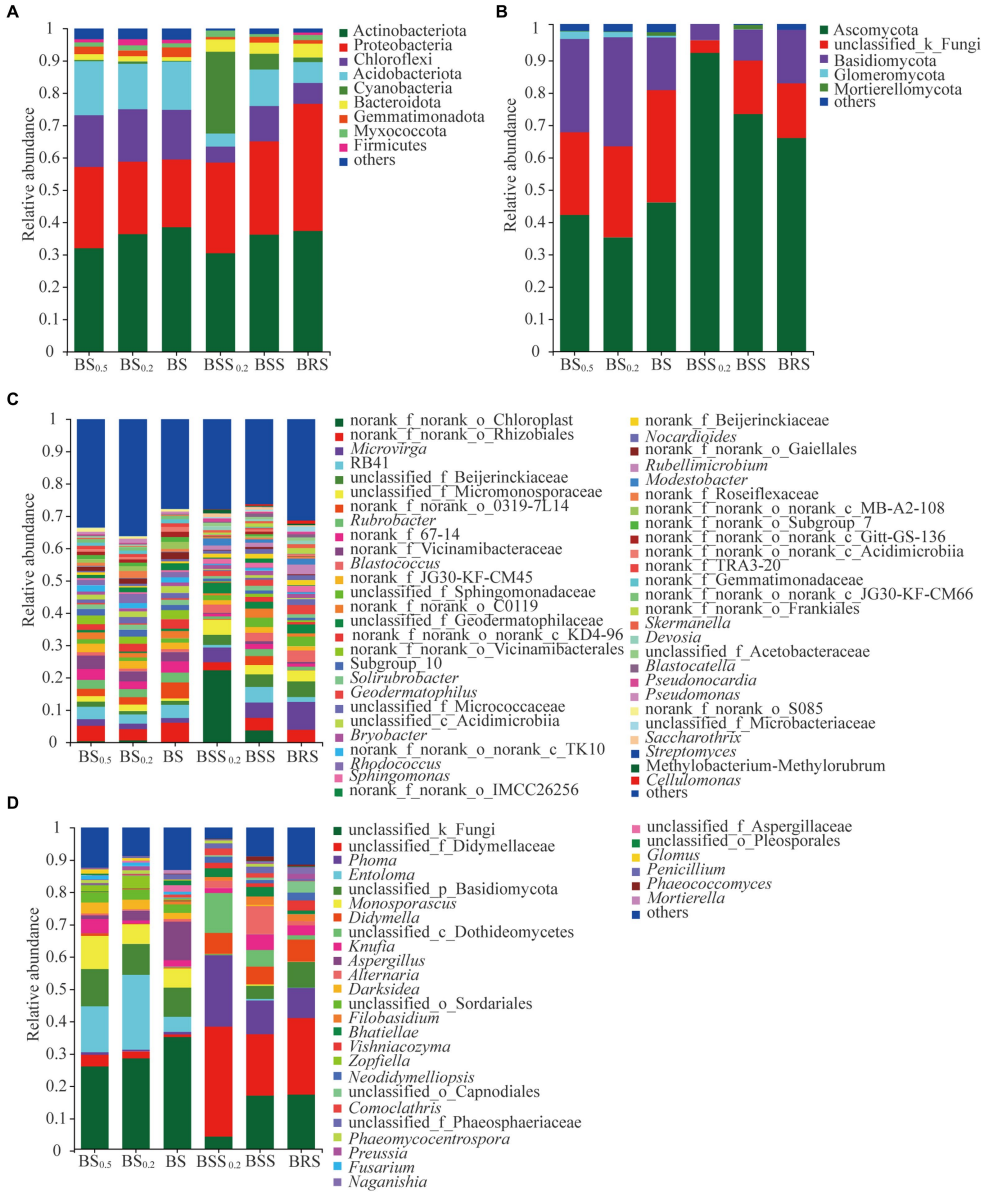
The community structure of bacteria and fungi. **(A)** Bacterial phyla composition. **(B)** Fungal phyla composition. **(C)** Bacterial genera composition. **(D)** Fungal genera composition. The meanings of BS_0.5_, BS_0.2_, BS, BSS, BSS_0.2_, and BRS are shown in Figure 1.

The structure of the soil bacterial and fungal communities at the phylum and genus levels is presented in Figure 5. The dominant phyla of bacterial community in BSS_0.2_, BSS, and BRS included Actinobacteriota, Protebacteria, Chloroflexi, Acidobacteriota, Acidobacteria, Cyanobacteria, and Bacteroidota (Figure 5A), collectively accounting for approximately 93%. Similarly, the dominant phyla of bacterial community in BS_0.5_, BS_0.2_, and BS consisted of Actinobacteriota, Protebacteria, Chloroflexi, Acidobacteriota, and Acidobacteria, representing approximately 90% of the community. On the other hand, Ascomycota and Basidiomycota were dominant phyla of fungal community in BBS_0.2_, BBS, and BRS (Figure 5B), comprising approximately 98% of the community. Similarly, in BS_0.5_, BS_0.2_, and BS, the dominant phyla of fungal community were Ascomycota, Basidiomycota, and Glomeromycota, accounted for approximately 95%. Additionally, the abundance of bacterial and fungal communities at the genus level in the six treated soil samples varied in the community structure (Figures 5C,D).

**Figure 6:**
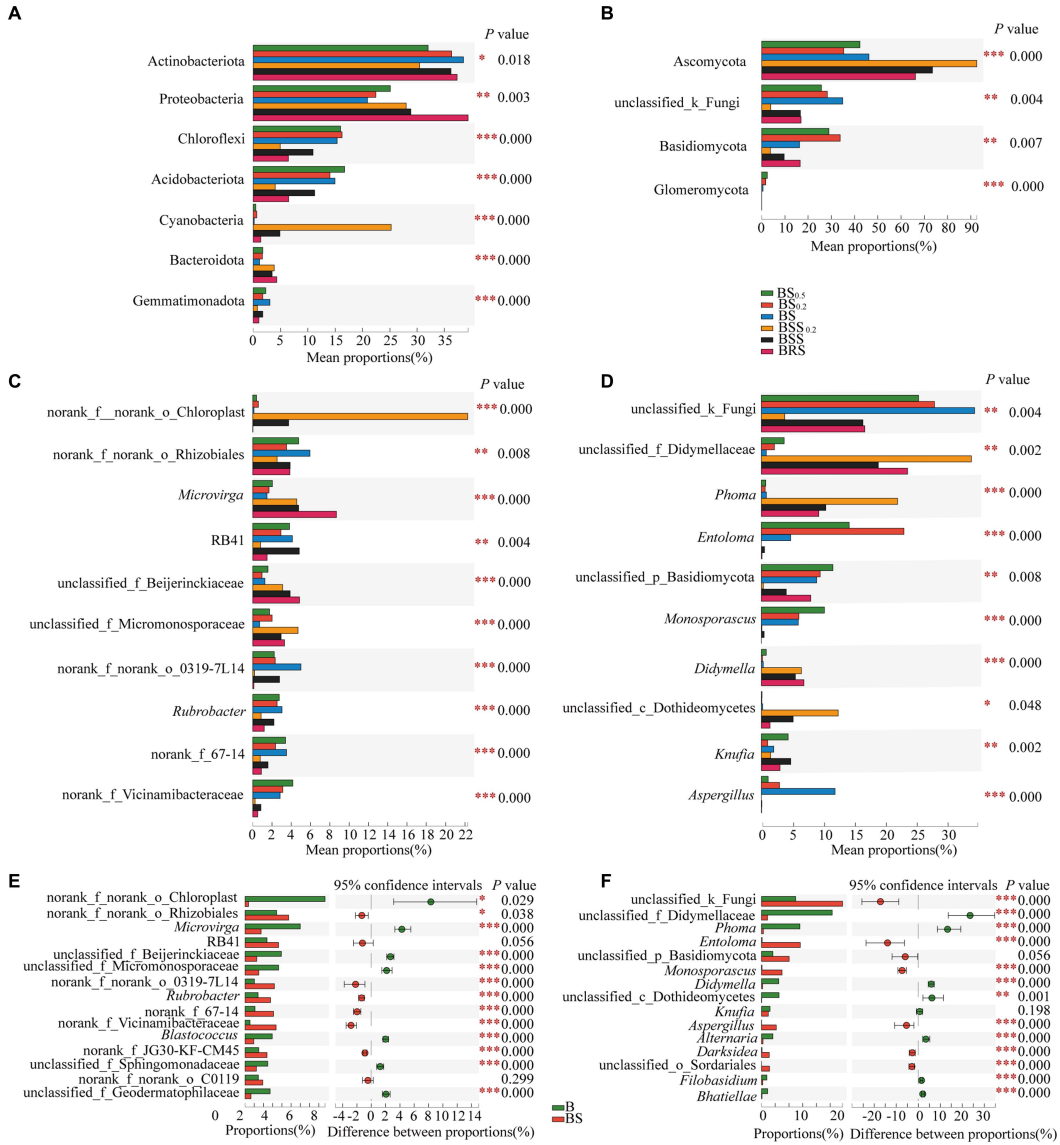
Comparison of community composition differences. **(A)** The differences of the relative abundance of important bacterial phyla between mossy biocrust rhizoid soil and different soil particle sizes of mossy biocrust sifting and sublayer soil. **(B)** The differences of the relative abundance of important fungal phyla between mossy biocrust rhizoid soil and different soil particle sizes of mossy biocrust sifting and sublayer soil. **(C)** The differences of the relative abundance of important bacterial genera between mossy biocrust rhizoid soil and different soil particle sizes of mossy biocrust sifting and sublayer soil. **(D)** The differences of the relative abundance of important fungal genera between mossy biocrust rhizoid soil and different soil particle sizes of mossy biocrust sifting and sublayer soil. **(E)** The differences of the relative abundance of important bacterial genera between mossy biocrust layer and sublayer. **(F)** The differences of the relative abundance of important fungal genera between mossy biocrust layer and sublayer. The meanings of BS_0.5_, BS_0.2_, BS, BSS, BSS_0.2_, and BAS are shown in Figure 1. * showed *p* < 0.05, ** *p* < 0.01, and *** *p* < 0.001.

The principle species and the differences in bacterial and fungal communities at the phylum levels are presented in Figures 6A,B. In bacterial communities (Figure 6A), the mean proportions of Actinobacteriota, Protebacteria, Chloroflexi, Acidobacteriota, Acidobacteria, Cyanobacteria, and Bacteroidota exhibited significant differences between BRS and different particle sizes of mossy biocrust sifting soil (BSS, BSS_0.2_) and sublayer soil (BS, BS_0.2_, and BS0_0.5_; *p* < 0.05). Notably, the mean proportions of Proteobacteria (39.35%) and Bacteroidota (4.31%) were the highest in BRS (*p* < 0.01). For the fungal community (Figure 6B), the mean proportions of Ascomycota, Basidiomycota, and Glomeromycota also demonstrated significant differences between BRS and mossy biocrust sublayer soil (BS, BS_0.2_, and BS0_0.5_; *p* < 0.01). Moreover, the mean proportion of Ascomycota (66.11%) was the highest in mossy biocrust sublayer soil (BS_0.5_, BS_0.2_, and BS).

The principle species and the differences in bacterial and fungal communities at the genus levels are presented in Figures 6C–F; the mean proportions of norank_f_norank_o_Chloroplast, *Microvirga*, norank_f_norank_o_Rhizobiales, RB41, *Rubrobacter*, unclassified_f_Beijerinckiaceae, unclassified_f_Micromonosporaceae, norank_f_Vicinamibacteraceae, norank_f_norank_o_0319-7 L14, and norank_f_67–14 exhibited significant differences in BRS, BSS, BSS_0.2_, BS, BS_0.2_, and BS0_0.5_ (*p* < 0.01). Among them, the mean proportions of *Microvirga* (8.67%) and unclassified_f_beijerinckiaceae (4.85%) in BRS were significantly higher than those in biocrust sublayer soil (BS, BS_0.2_, and BS0_0.5_) and mossy biocrust sifting soil (BSS, BSS_0.2_; *p* < 0.01). In the fungal community, there were also significant differences at the genus level (*p* < 0.01) among fungal communities of the six differently treated soils (Figure 6D). For example, the mean proportions of unclassified_ f_didymellaceae, *Phoma*, *Entoloma*, *u*nclassified_p_Basidiomycota, *Monosporascus*, *Didymella*, *Knufia*, *Aspergillus*, and *Phoma* showed significant differences in BRS, BSS, BSS_0.2_, BS, BS_0.2_, and BS0_0.5_ (*p* < 0.01). Furthermore, various other genus level differences are detailed, highlighting the distinctions in community composition across the different soil treatments.

Overall, the detailed analysis provided various insights into the composition and structure of the bacterial and fungal communities at both phylum and genus levels, offering valuable information for understanding the microbial dynamics in the studied soils.

### 3.5 Microbial co-occurrence networks in mossy biocrust

**Figure 7:**
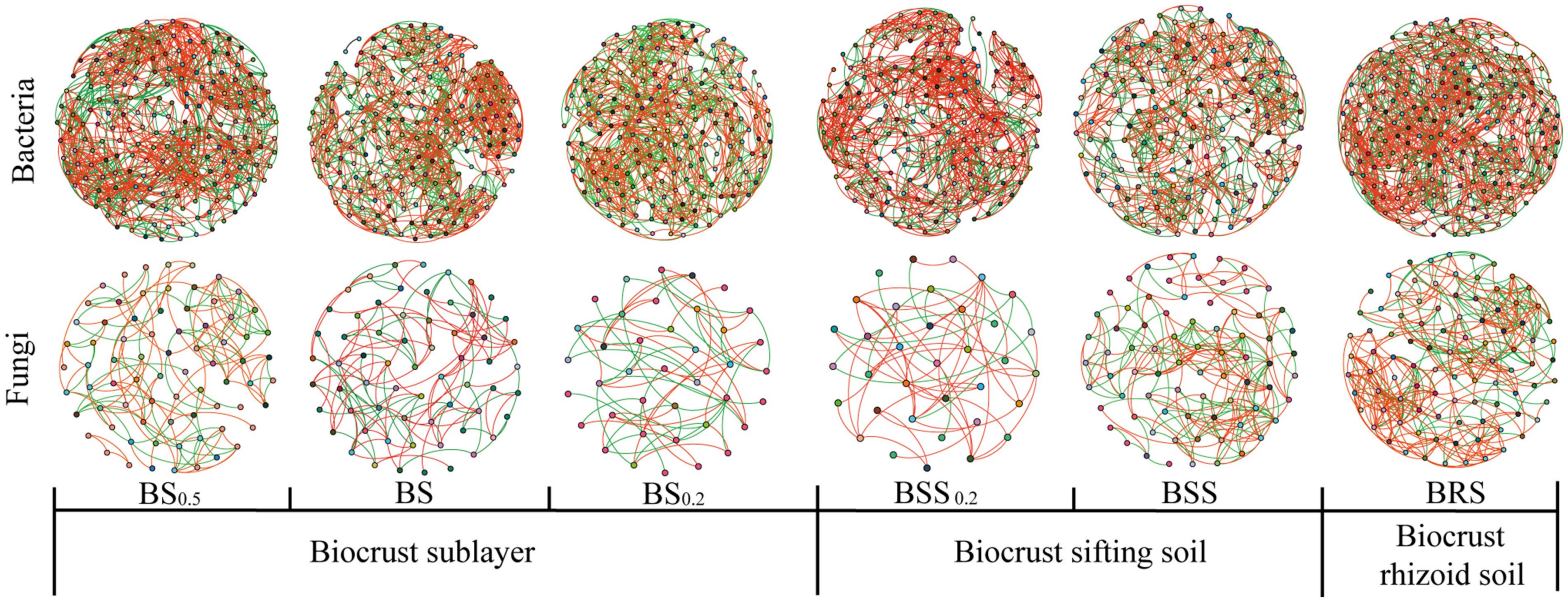
Network visualization of microbial co-occurrence. The meanings of BS_0.5_, BS_0.2_, BS, BSS, BSS_0.2_, and BRS are shown in Figure 1.

In the bacterial community network co-occurrence diagram, both the mossy biocrust sublayer soils (BS, BS_0.2_, and BS_0,5_) and the mossy biocrust layer soil (BSS, BSS_0.2_, and BRS) exhibited a higher percentage of positive links compared with negative links. Conversely, in the fungal network co-occurrence, the percentage of positive links was higher in the mossy biocrust layer soil than mossy biocrust sublayer soil (Table 2; Figure 7). Notably, among the six different soil treatments, BRS demonstrated the highest percentage of positive links (Figure 7). As per the information presented in Table 2, in the network co-occurrence of bacteria, the number of edges and nodes in biocrust sublayer soil (BS_0.5_, BS_0.2_, and BS) was higher than those in biocrust sifting soil (BSS_0.2_, BSS). Moreover, BRS exhibited the highest number of edges and nodes in both the fungal and bacterial co-occurrence networks. Compared with BS_0.5_, BS_0.2_, BS, BSS, and BSS_0.2_, the BRS demonstrated the best complexity and connectivity of bacterial and fungal networks.

**Table 2:** Property of microbial network.

	Property	BS_0.5_	BS_0.2_	BS	BSS_0.2_	BSS	BRS
Bacterial	Edge	1,197	1,073	1,112	1,004	816	1,394
	Note	190	188	188	173	158	214
	Percentage of positive links	54.3	50.42	52.57	63.15	52.21	53.16
	Average clustering coefficient (%)	0.551	0.553	0.570	0.563	0.576	0.568
	Density	0.067	0.061	0.063	0.067	0.061	0.066
	Average degree	12.6	11.415	11.83	11.607	10.29	13.028
Fungi	Edge	158	66	137	59	221	361
	Note	74	39	63	35	75	94
	Percentage of positive links (%)	56.96	51.52	62.04	77.97	54.75	65.65
	Average clustering coefficient	0.552	0.775	0.591	0.349	0.542	0.62
	Density	0.058	0.089	0.07	0.08	0.099	0.083
	Average degree	4.27	3.385	4.349	3.371	5.893	7.681

## 4 Discussion

### 4.1 The impact of mossy biocrust rhizoid and sifting soil on soil surface environment

In our study, we observed that the organic carbon (TOC), total nitrogen (TN), and total phosphorus (TP) in the mossy biocrust sifting soil were significantly higher compared with the biocrust sublayer soil. This finding is consistent with previous studies that have reported the elevated levels of organic matter, TN, total carbon, total nitrogen, total phosphorus, nitrate nitrogen, and ammonium nitrogen in mossy crust soil compared with mossy crust subsoil (Chamizo et al., 2012; Zhang et al., 2022). In arid desert environment with minimal rainfall, the presence of mossy crust microorganisms (biological residues) has been shown to effectively increased soil organic matter content (Xu et al., 2022; Zhang et al., 2022). Furthermore, in our study, we found that the TOC, TN, NH_4_^+^, and NO_3_^−^ in the mossy biocrust rhizoid soil were significantly higher than those in both the mossy biocrust sifting soil and the mossy biocrust sublayer soil, and this indicates that mossy biocrust rhizoid soil effectively enhanced the nitrogen supply capacity of the soil. Although moss itself does not fix nitrogen, as the crust develops, the composition of the bacterial community undergoes changed, and the increase in RB4l (Acidobacteria) and uncultured_bacterium_f Longimicrobiaceae (Gemmatimonadetes) has been found to replace some cyanobacteria in the process of fixing carbon and nitrogen (Pietrasiak et al., 2013; Guang et al., 2023). Additionally, microbial necromass has been identified as a significant source of organic matter in sandy soils dominated by biological crusts and contributes to soil organic carbon (SOC) (Wang et al., 2022). These factors might contribute to the higher nitrogen fixation in BRS than that in mossy biocrust sifting soil and mossy biocrust sublayer soil. Previous studies have demonstrated that NH_4_^+^ and NO_3_^−^ in the subsoil of mossy biocrust exert a substantial influence on the nitrogen content of moss (Li et al., 2023), and it is suggested that mossy crust could primarily utilize NH_4_^+^ in the soil through the rhizoid as the main nitrogen source.

### 4.2 Extracellular enzyme activities and microbial nutrient limit in mossy biocrust

The enzyme activities of mossy biocrust were found to be higher than those in mossy crust sublayer soil, bare sand, and other biocrust succession stages (Zhang et al., 2022). Additionally, our study revealed that the activity of C-acquisition enzyme (BG) in BRS was significantly higher than that in BSS and BS, leading to significantly higher C: P and C: N enzyme activities in BRS compared with BSS and BS. Energy limitation is common for soil microbial communities in ultra-oligotrophic desert ecosystem (Tapia-Torres et al., 2015; Moorhead et al., 2016), and microbial nitrogen limitation is also common in biocrust (Wang et al., 2022), which aligns with our own research findings. Moreover, we observed that BRS exhibited the highest levels of C and N limitation. On one hand, plant litter, including mossy litter, is known to be difficult to decompose and utilize by microorganisms, hence leading to the limitation of soil microorganism C (Williams et al., 1998; Maksimova et al., 2013; Cui et al., 2019; Li et al., 2023). Similarly, the slow decomposition of moss, particularly in a sandstorm environment (Philben et al., 2018), allows C and N to persist in the litter bank for extended periods. This situation results in the production of more C-acquisition enzymes by microorganisms in the mossy biocrust rhizoid soil being limited by the maximum relative C. On the other hand, a study has demonstrated that the microbial necromass significantly contributes to soil organic carbon accumulation during initial soil formation in biocrust-dominated surfaces, and this process leads to an increase in N-acquiring enzyme activities with biocrust formation, accelerating necromass decomposition and alleviating microbial N limitation during the initial soil formation (Wang et al., 2022). Therefore, it is possible that microbial necromass decomposition in mossy biocrust has a significant impact on microbial N limitation.

### 4.3 Spatial differentiation of microbial composition in mossy biocrust

In our research, we found that the fungal community richness in the mossy boicrust layer soil was significantly higher than that in the mossy biocrust sublayer soil, which aligns with the previous research results that the fungal microbial richness in the mossy crust is significantly higher than that in the lower layer of mossy crust in the Loess Plateau of China (Xiao and Veste, 2017). Furthermore, we observed that the fungal community diversity and richness in BRS were significantly higher than that in the mossy biocrust sublayer soil. The rhizoid effect of mycorrhiza in the mossy biocrust rhizoid soil may contribute to the increase in fungal diversity in the rhizosphere (Zhang et al., 2017; Ye et al., 2021). Plants are known to influence the beneficial members of the rhizosphere microbial community through specific components in rhizosphere secretions (Ling et al., 2022; Sokol et al., 2022). Additionally, biological soil crusts provide an optimized environment for crust soil microorganisms through the secretion of extracellular polymeric substances (EPS) and primary production (Meier et al., 2021), which enables mossy crusts to establish favorable microhabitats with sufficient water, moderate temperature, and rich living nutrition in harsh environment (Xiao and Veste, 2017). This may be one of the reasons why the Sobs index and Chao 1 index of α-diversity species richness of fungal communities in BRS were higher than those in mossy biocrust sifting soil and mossy biocrust sublayer soil.

We also identified the dominant bacteria in the mossy biocrust layer soil, including Actinobacteriota, Proteobacteria, Chloroflexi, Acidobacteriota, Acidobacteria, Cyanobacteria, and Bacteroidota, with a similar dominant bacteria phylum found in the mossy crust subsoil. Furthermore, Ascomycota and Basidiomycota were determined to be the important dominant fungi in mossy biocrust layer soil, consistent with previous research results in the Gurbantunggut Desert (Liu et al., 2019). We observed a higher relative abundance of Proteobacteria and Bacteroidota in BRS, indicating their enrichment in the mossy biocrust rhizoid and highlighting them as core microorganisms in BRS. Our findings align with previous research (Ling et al., 2022), indicating that Bacteroidea and Proteobacteria were enriched in the rhizosphere; Proteobacteria and Bacteroidota are generally adapted to C-rich conditions, demonstrating high metabolic activity, fast growth, and propagate (Pausch et al., 2013; Kuzyakov and Razavi, 2019). Moreover, the high relative abundance of Bacteroidota in BRS indicates their potential to degrade polymeric polysaccharides by biological soil crust, while the higher relative abundance of Proteobacteria in mossy biocrust rhizoid soil suggests their involvement in the high-frequency nutrient exchange and rhizosphere secretion. Furthermore, our observations indicated that the relative abundance of Proteobacteria in mossy biocrust rhizoid soil was the highest. This could be attributed to the soil’s high capacity for fixing carbon and nitrogen. BRS is a component of the rhizosphere secretion area and facilitates high-frequency nutrient exchange, potentially contributing to the elevated relative abundance of Proteobacteria in BRS. Additionally, the relative abundance of Ascomycota in mossy biocrust layer soil was significantly higher than that in mossy biocrust sublayer soil, suggesting their involvement in the degradation of lignin and recalcitrant carbon in stable biological soil crusts. This indicates that fungi may have a more restrictive role than bacteria and plants in influencing arid land ecological processes (Lienhard et al., 2013; Liu et al., 2018; Zhao et al., 2020).

These findings provide valuable insight into the intricate microbial dynamics within biological soil crusts, particularly in relation to fungal and bacterial community diversity and richness, shedding light on their potential roles in nutrient cycling and ecological stability.

### 4.4 Microbial network complexity and stability in mossy biocrust

Zhou et al. demonstrated that the complexity and connectivity of the bacterial and fungal networks in moss crust increased with the continuous development of biological soil crusts in Mu Us sandy land in northwest China (Zhou et al., 2020). Additionally, Zhang et al. found that the bacterial and fungal network structures in the biocrust layer were more complex than biocrust sublayer in subtropical karst ecosystem (Zhang et al., 2022). However, a study (Guang et al., 2023) revealed that the network edges and notes of mossy crust subsoil in the Tengger Desert were higher than those in mossy crust subsoil. Our study further found that compared with mossy biocrust sifting soil and mossy biocrust sublayer soil, BRS exhibited the highest complexity and best connectivity.

The shift in dominance bacteria changes competition and cooperation between species in the bacterial community, impacting the complexity and stability of the bacterial network (De Vries et al., 2018). Additionally, the rhizosphere regulates microbe-facilitated soil processes and services (Bhattacharyya and Furtak, 2023), with plant root-associated secretions playing a key role in the rhizosphere by affecting the growth of microorganisms (Fan et al., 2017). Moreover, previous studies have indicated that biological soil crust has the ability to produce extracellular polysaccharides secreted by filamentous cyanobacteria (Belnap et al., 2016). As a producer, moss supplied organic carbon and other resources for the soil microbial community (Liu et al., 2018). It is worth considering whether BRS can also produce extracellular polysaccharides. The stability of mossy crust during the succession of biological soil crust succession may be attributed to the physical and chemical properties of the rhizoid soil, sifting soil, and sublayer soil of mossy crust. We discovered that the TOC, TN, and NO_3_^−^ in BRS were significantly higher than those in mossy biocrust sifting soil and sublayer soil. Additionally, TP and NH_4_^+^ in BRS were significantly higher than those in mossy biocrust sublayer soil, and NH_4_^+^ in BRS was significantly higher than that in mossy biocrust sifting soil. These findings may help to explain why co-occurrence network of bacteria and fungi communities in BRS was more complicated and better connected.

## 5 Conclusion

BRS showed higher C-degrading enzyme activity and lower P-degrading enzyme activity. Mossy biocrust soils were all limited by microbial relative N, with strong relative N limitation and microbial maximum relative C limitation in BRS. There were significant differences in bacterial and fungal community species composition in the mossy biocrust and sublayer with different particle sizes. The bacterial and fungal communities displayed significant differences at the mean proportions of important phylum and genus levels between BRS and different particle sizes of mossy biocrust sifting soil and mossy biocrust sublayer soil. The highest complexity connectivity of bacterial and fungal network in BRS was higher than that of different particle sizes of mossy biocrust sifting soil and mossy biocrust sublayer soil.

## Data availability statement

The datasets presented in this study can be found in online repositories. The names of the repository/repositories and accession number(s) can be found at: https://www.ncbi.nlm.nih.gov/, SRP438853.

## Author contributions

XD: Data curation, Formal analysis, Investigation, Writing – original draft, Software. JiaL: Investigation, Software, Writing – original draft. WH: Data curation, Investigation, Writing – original draft. JH: Data curation, Investigation, Writing – original draft. WX: Data curation, Investigation, Writing – original draft. SC: Data curation, Investigation, Writing – original draft. SL: Data curation, Investigation, Writing – original draft. JianL: Resources, Software, Writing – review & editing. XZ: Resources, Software, Writing – review & editing. JinL: Investigation, Methodology, Project administration, Supervision, Writing – review & editing, Funding acquisition.
